# Understanding the action mechanisms of metformin in the gastrointestinal tract

**DOI:** 10.3389/fphar.2024.1347047

**Published:** 2024-03-28

**Authors:** Meihui Cheng, Lili Ren, Xianxian Jia, Jianwei Wang, Bin Cong

**Affiliations:** ^1^ Research Unit of Digestive Tract Microecosystem Pharmacology and Toxicology, National Institute of Pathogen Biology, Chinese Academy of Medical Sciences and Peking Union Medical College, Beijing, China; ^2^ College of Forensic Medicine, Hebei Key Laboratory of Forensic Medicine, Collaborative Innovation Center of Forensic Medical Molecular Identification, Hebei Medical University, Shijiazhuang, China; ^3^ NHC Key Laboratory of Systems Biology of Pathogens and Christophe Mérieux Laboratory, National Institute of Pathogen Biology, Chinese Academy of Medical Sciences and Peking Union Medical College, Beijing, China; ^4^ Department of Pathogen Biology, Institute of Basic Medicine, Hebei Medical University, Shijiazhuang, China

**Keywords:** drug mechanism, metformin, type 2 diabetes, gastrointestinal tract, gut microbiota

## Abstract

Metformin is the initial medication recommended for the treatment of type 2 diabetes mellitus (T2DM). In addition to diabetes treatment, the function of metformin also can be anti-aging, antiviral, and anti-inflammatory. Nevertheless, further exploration is required to fully understand its mode of operation. Historically, the liver has been acknowledged as the main location where metformin reduces glucose levels, however, there is increasing evidence suggesting that the gastrointestinal tract also plays a significant role in its action. In the gastrointestinal tract, metformin effects glucose uptake and absorption, increases glucagon-like peptide-1 (GLP-1) secretion, alters the composition and structure of the gut microbiota, and modulates the immune response. However, the side effects of it cannot be ignored such as gastrointestinal distress in patients. This review outlines the impact of metformin on the digestive system and explores potential explanations for variations in metformin effectiveness and adverse effects like gastrointestinal discomfort.

## 1 Introduction

Metformin originates from the herbaceous plant *Galega officinalis*, commonly referred to as *French lilac* or *goat’s rue* in Europe (Bailey and Day, 1989). In the early 1900s, scientists identified the guanidine compounds and related molecules, rich in *G. officinalis*, as the basis for their biological action and glucose-lowering properties (Werner and Bell, 1922). Metformin was initially created in 1922 by *Emil Werner* and *James Bell*. Further research demonstrated its notable ability to lower blood sugar levels in animal experiments, including rabbits (Bailey, 2017; Triggle et al., 2022). However, because of the discovery of insulin, metformin research was put on hold. It was not until the 1950s that *Jean Sterne* demonstrated the effectiveness of metformin in treating diabetes in human trials for the first time (Sterne, 1963). With his efforts, there were more comprehensive basic and clinical studies on metformin. Metformin’s role has been increasingly acknowledged by different nations, making it the preferred medication for managing type 2 diabetes mellitus (T2DM) in clinical settings (Davies et al., 2018).

In addition to treating T2DM, metformin modestly reduces body weight gain, which may be achieved by upregulating growth differentiation factor 15 (GDF15) (Day et al., 2019; Coll et al., 2020). Notably, Notably, metformin also has protective and therapeutic effects against COVID-19 (Bramante et al., 2021; Khunti et al., 2021; Lalau et al., 2021). A study published recently found that the use of metformin during the initial phases of a COVID-19 infection decreased the likelihood of developing Long COVID (Bramante et al., 2022). Besides, metformin may also have a therapeutic effect on other diseases, such as cancer (Evans et al., 2005; Thakkar et al., 2013; Foretz et al., 2014; Heckman-Stoddard et al., 2017), inflammation-related diseases (Kalender et al., 2010), and even against aging (Valencia et al., 2017; Kulkarni et al., 2020). Nevertheless, the main clinical application of metformin is the treatment of T2DM.

Initial research typically viewed the liver as the main location where metformin exerts its control over glucose production, utilizing both AMP-activated protein kinase (AMPK)-dependent and AMPK-independent pathways. Nevertheless, increasing proof indicates that metformin primarily acts on the gastrointestinal system. Intravenous metformin was found to be less glucose-lowering than oral administration (Stepensky et al., 2002). In addition, metformin concentrations in the jejunum peaked at 500 μg/g, 30–300 times higher than that in plasma (McCreight et al., 2016). Metformin inhibits dietary glucose absorption from the gastrointestinal tract in rodents (Wilcock and Bailey, 1991; Ikeda et al., 2000; Wu et al., 2017) and T2DM patients (Bailey, 1995). Positron emission computed tomography (PET-CT) imaging confirmed this discovery. After infusion of ^18^F-labeled fluorodeoxyglucose (^18^F-FDG), which is a non-metabolisable glucose analogue, into the intestinal lumen and sealing of the compartment system through surgery, PET-CT imaging showed accumulation of ^18^F-FDG in the intestinal lumen of diabetic rats receiving a single oral dose of metformin (Zubiaga et al., 2023). One of the possible reasons for this is that metformin inhibits sodium-glucose cotransporter1 (SGLT1) on small intestinal epithelial cells, decreasing the intra- and extracellular Na^+^ concentration gradient, thereby inhibiting intestinal glucose absorption and increasing glucose utilization (Lenzen et al., 1996; Gorboulev et al., 2012). Apart from this, metformin can cause changes in the microbial composition of the intestinal tract (Vallianou et al., 2019), increased lactic acid production (Misbin et al., 1998), and increased concentrations of glucagon-like peptide-1 (GLP-1) and bile acids (Mannucci et al., 2004; Brønden et al., 2017), but the mechanism by which metformin acts in the gastrointestinal tract is currently not precise.

Despite its widespread availability and affordability (Foretz et al., 2014; Matthews et al., 2019; Ahmad et al., 2020; Schernthaner and Schernthaner, 2020), metformin is associated with gastrointestinal side effects in around 20% of patients, including nausea, vomiting, diarrhea, bloating, and occasionally lactic acidosis and vitamin B12 malabsorption. (Sanchez-Rangel and Inzucchi, 2017; Schommers et al., 2017). Approximately 5% of individuals stopped taking the medication because of negative reactions (Sanchez-Rangel and Inzucchi, 2017; Schommers et al., 2017). However, the reasons for differences in metformin side effects are unclear. In this article, we will review the progress of research effects of metformin on the gastrointestinal tract in recent years in the hope of shedding light on future studies of metformin treatment and side effect mechanisms.

## 2 Effects of metformin on the gastrointestinal tract

### 2.1 Effects on glucose uptake and absorption

PET-CT imaging-based studies have found that metformin modulates glucose in the intestine (Chang et al., 2020). By using ^18^F-FDG as a contrast agent, PET-CT technology was used to reflect changes in body metabolism through the uptake of the contrast agent by the lesion, where ^18^F-FDG is a glucose analog that indicates the degree of glucose uptake and is generally used clinically to quantify tissue metabolism (Paydary et al., 2019). Using PET-CT imaging after intravenous administration of ^18^F-FDG to T2DM patients treated with metformin, researchers showed that the amount of glucose entering the gastrointestinal system from the circulation was increased, which contributed to the drug’s glucose-lowering effect and improved glycemic control (Koffert et al., 2017; Chang et al., 2020; Tobar et al., 2023). Likewise, PET-CT scans of mice fed a high-fat diet (HFD) showed that metformin increased glucose uptake on the basolateral side of the intestine, leading to enhanced glucose tolerance in a manner that depended on the dosage (Tobar et al., 2023). This indicates that metformin controls the movement of glucose from the bloodstream to the intestines.

Mechanistically, metformin inhibits intestinal glucose absorption (Wilcock and Bailey, 199). The inhibition of intestinal glucose absorption is due to reduce the expression of SGLT1 on the apical membrane of jejunal enterocytes (Zubiaga et al., 2023). In mice lacking SGLT1, the reduction in postprandial glucose response mediated by a single administration of metformin is attenuated, but not in mice lacking glucose transporter 2 (GLUT2). This suggests that metformin-induced glucose reduction is dependent on SGLT1 expression (Zubiaga et al., 2023). In addition, metformin also lowers blood glucose by enhancing intestinal glucose uptake. After being consumed in the intestines, glucose is moved to the intestinal lumen and expelled through the faeces, helping to regulate blood sugar levels. Based on the high accuracy and contrast of positron emission computed tomography-magnetic resonance imaging (PET-MRI), it was found that metformin-induced ^18^F-FDG accumulation in the intestinal wall as well as in the lumen of the ileum and colon of T2DM patients treated with metformin in a dose-dependent manner (Morita et al., 2020; Ito et al., 2021). This indicates that metformin may enhance glucose release from enterocytes into the lumen.

GLUT2 protein moves from the basolateral membrane of the small intestine to the apical membrane of the jejunum to aid in glucose uptake when the small intestine’s intestinal lumen has high levels of glucose (Lostao et al., 1991). Notably, metformin increases GLUT2 expression on the jejunal parietal membrane (Lenzen et al., 1996). Contrary to the findings of this research, it was discovered that metformin therapy had no effect on the expression of the GLUT2 gene in enterocytes (Lenzen et al., 1996). Furthermore, the impact of metformin on glucose uptake in the digestive system is associated with the amount of GLUT2 expression, rather than relying on GLUT2 expression in the intestinal lining (Morrice et al., 2023). However, metformin has been shown to enhance the translocation of GLUT2 to the apical membrane of enterocytes (Ait-Omar et al., 2011), providing a potential mechanism for the release of glucose from enterocytes into the intestinal lumen in response to metformin treatment (Ke et al., 2023). Notably, mutations in the *GLUT2* gene affect the glucose-lowering efficacy of metformin (Zhou et al., 2016), which may be one of the reasons for individual differences in metformin efficacy. Although studies have found that metformin also affects ^18^F-FDG accumulation in the colon, the mechanism is unclear (Ito et al., 2021). In conclusion, these studies suggest that metformin can achieve its glucose-lowering ability by modulating glucose transporter proteins in the gut.

### 2.2 Increased GLP-1 secretion

Metformin enhances the release of glucagon-like peptide-1 (GLP-1) from the intestines, potentially contributing to its ability to lower blood sugar levels (Mannucci et al., 2004; McCreight et al., 2016; Bahne et al., 2018). GLP-1, a hormone produced by L cells in the jejunum, ileum, and colon, is released in response to nutrients (D’Alessio, 2016). It interacts with its receptor, causing a rise in cyclic adenosine monophosphate (cAMP) levels, which stimulates the secretion of insulin from pancreatic β-cells (Bradley et al., 2010; D’Alessio, 2016). Additionally, it has the ability to decrease the overproduction of glucagon in response to glucose levels (Bradley et al., 2010; D’Alessio, 2016). Thus, when blood glucose increases, GLP-1 stimulates insulin secretion while decreasing glucagon secretion; when blood glucose decreases, GLP-1 inhibits insulin secretion without affecting glucagon secretion.

The mechanism by which metformin increases GLP-1 secretion in the gastrointestinal tract is currently unclear. Commonly believed mechanisms for the enhancement of GLP-1 secretion are generally considered to be the following:

(1) Metformin boosts the absorption of glucose in the upper section of the small intestine by enhancing the production of glucose transporter proteins in the epithelial cells of the small intestine. This results in a rise in glucose levels in the end part of the intestine, which then triggers an increase in the release of GLP-1 from L cells, promoting growth (Wu et al., 2017).
(2) Metformin increases the concentration of GLP-1 in the gastrointestinal tract by inhibiting the activation of dipeptidyl peptidase-4 (DPP4), which in turn reduces the degradation of GLP-1 by DPP4 in the gastrointestinal tract (McCreight et al., 2016). In mice and humans taking metformin, a decrease in DPP4 levels is observed (Lenhard et al., 2004; Green et al., 2006; Cuthbertson et al., 2009). However, *in vitro* experiments, metformin did not directly inhibit DPP4 activity (Hinke et al., 2002). Therefore, metformin may not act directly on DPP4. It has been found that after knocking out the *DPP4* gene in rats, metformin could still dose-dependently increase GLP-1 concentrations (Yasuda et al., 2002). In line with this finding, the inclusion of a DPP4 blocker alongside metformin led to a greater rise in GLP-1 levels, indicating synergistic and overlapping impacts, implying distinct modes of action for each drug (Migoya et al., 2010). Thus, metformin increases the concentration of GLP-1 *in vivo*, probably not primarily through inhibition of DPP4.
(3) Metformin may also boost GLP-1 release in the gut by hindering bile acid reabsorption and raising bile acid levels, indirectly triggering GLP-1 secretion from L cells. Research has shown that metformin can block the reabsorption of bile acids by reducing the levels of Na^+^-dependent bile acid transporter proteins and controlling the function of farnesoid X receptor (FXR) (Lien et al., 2014; Foretz et al., 2019). Metformin is commonly thought to block FXR function through an AMPK-mediated process, resulting in reduced bile acid absorption in the ileum. This leads to increased bile acids in the intestinal lumen, which then trigger Takeda G-protein-coupled receptor 5 (TGR5) on L-cells, stimulating the production and release of GLP-1 (Thomas et al., 2009; Lien et al., 2014). Recent research has discovered that metformin can inhibit FXR signaling by altering gut bacteria, leading to increased GLP-1 production and release in a way that does not depend on AMPK activation (Sun et al., 2018). For example, metformin-treated T2DM patients with elevated levels of *Bacteroides fragilis* in the gastrointestinal tract, whose metabolite glycoursodeoxycholic acid (GUDCA) is involved in inhibiting FXR signaling (Sun et al., 2018). Therefore, regulating gut microbiota may also be involved in GLP-1 production and secretion.
(4) Metformin may enhance GLP-1 secretion through PEN2, a subunit of γ-secretase. A recent study found that the ability of metformin to promote GLP-1 secretion was impaired in mice with a gut-specific knockout of the PEN2 gene (Ma et al., 2022). This study discusses how low-dose metformin activates AMPK without altering AMP/ADP levels, contrary to the traditional understanding of its action. The researchers identified PEN2 as a metformin binding partner that forms a complex with ATP6AP1, leading to AMPK activation through inhibition of the lysosomal v-ATPase. And this process does not affect cellular energy levels (AMP/ADP ratios). Additionally, when PEN2 is specifically knocked out in the gut, the ability of metformin to promote insulin and GLP-1 secretion is impaired (Ma et al., 2022). Therefore, PEN2 plays a key role in the mechanism by which metformin enhances GLP-1 secretion.
(5) Metformin may also enhance GLP-1 secretion via the M3 cholinergic receptor and the gastrin-releasing peptide (GRP) receptor. The researchers found that the mechanism by which metformin stimulates GLP-1 release involves two G-protein-coupled receptors, the M3 cholinergic receptor and the GRP receptor (Mulherin et al., 2011). By pre-treating rats with the non-specific cholinergic receptor antagonist atropine as well as the specific M3 cholinergic receptor antagonist 4-DAMP and performing vagotomy, the researchers found that atropine and 4-DAMP significantly reduced metformin-induced GLP-1 secretion, whereas vagotomy had no effect on metformin-induced GLP-1 secretion. This indicates that metformin promotes GLP-1 release through a non-vagal M3 muscarinic pathway (Mulherin et al., 2011). Additionally, by pre-treating with the GRP receptor antagonist RC-3095, they found that GRP also plays a partial role in metformin-induced GLP-1 secretion. Therefore, M3-type cholinergic receptors and GRP receptors are also involved in metformin-induced changes in GLP-1 secretion.

In summary, metformin can promote GLP-1 secretion in the gastrointestinal tract through several mechanisms. Despite metformin’s ability to lower glucose levels by boosting GLP-1 secretion in living organisms, it did not have the same effect on GLP-1 secretion in L cells during laboratory experiments (Mulherin et al., 2011). Furthermore, research has indicated that blocking or lack of enteric glucagon receptor signaling, like glucagon-like peptide-1 receptor (GLP-1R), does not entirely eliminate the positive impacts of metformin on the blood sugar response to oral glucose in healthy or overweight mice (Maida et al., 2011). Therefore, increasing GLP-1 secretion may not be the main mechanism through which metformin reduces blood sugar levels.

### 2.3 Alteration of the gut microbiota

With the emergence of advanced sequencing technologies, it has become apparent that metformin mitigates the imbalance in gut microbiota associated with T2DM and its subsequent impact on host metabolism. Metformin is believed to cause substantial changes in the composition and operation of the intestinal microbiome, as stated by several studies (Forslund et al., 2015; McCreight et al., 2016; Wu H. et al., 2017; Vallianou et al., 2019; Zhang and Hu, 2020; Jones and Molloy, 2021; Mueller et al., 2021). Several research studies with rodents fed a high-fat diet, different animal models of obesity and diabetes, and patients diagnosed with T2DM have all shown changes in gut microbiota caused by metformin, as shown in Table 1. Notably, the diversification of gut microbiota resulting from metformin treatment was observed following oral administration in mice, a phenomenon not replicated with intraperitoneal injection (Broadfield et al., 2022). This finding aligns with the observed enhancement of glucose regulation in high-fat diet mice receiving fecal transplants from metformin-treated counterparts (Wu H. et al., 2017; Bauer et al., 2018). The elimination of gut bacteria with antibiotics canceled out metformin’s ability to reduce glucose levels in mice fed a high-fat diet (Shin et al., 2014), highlighting the importance of gut bacteria in how metformin works.

**TABLE 1:** The effects of metformin on the composition of gut microbiota.

Research object	Detection method	Effect		References
HFD C57BL/6 mice	16S rRNA	up	*Akkermansia, Alistipes*	Zhang and Hu (2020)
		down	*Anaerotruncus*, *Lactococcus, Parabacteroides, Odoribacter, Lawsonia, Blautia, Lactonifactor*	
HFD C57BL/6 mice	16S rRNA	change	*Bacteroidaceae*,* Verrucomicrobiaceae*, *Akkermansia muciniphila, Clostridium cocleatum*	Lee and Ko (2014)
db/db mice	16S rRNA	up	*Bacteroidales, Lactobacillus, Allobaculum, Bacteroides, Akkermansia*	Chen et al. (2018)
		down	*Staphylococcus, Corynebacterium, Jeotgalicoccus, Aerococcus, Enterococcus, Facklamia*	
HFD C57BL/6 mice	16S rRNA	up	*Verrucomicrobia, Akkermansia, Bacteroides, Butyricimonas, Parabacteroides*	Lee et al. (2018)
		down	*Firmicutes/Bacteroidetes* ratio	
HFD C57BL/6 mice	16S rRNA	up	*Akkermansia, Bifidobacterium pseudolongum*	Zheng et al. (2018)
		down	*Firmicutes*	
db/db mice	16S rRNA	up	*Butyricimonas, Lactobacillus, Coprococcus, Ruminococcus, Akkermansia*	Zhang et al. (2019)
		down	*Prevotella, Proteus*	
HFD C57BL/6 mice	16S rRNA	up	*Bacteroides, Akkermansia, Parabacteroides, Christensenella*	Ryan et al. (2020)
		down	*Muribaculum, Lachnoclostridium, Coprococcus, Dorea, Papillibacter, Oscillospira, Ruminococcus, Desulfovibrio,* Desulfovibrionaceae	
HFD Wistar rats	16S rRNA	up	*Akkermensia, Allobaculum, Bacteroides, Blautia, Butyricicoccus, Lactobacillus, Phascolarctobacterium, Parasutterella, Klebsiella, Prevotella*	Zhang et al. (2015)
		down	*Clostridium XIVa, Flavonifractor, Lachnospiracea_incertae_sedis, Roseburia, Clostridium XI*	
HFD Wistar rats	16S rRNA	up	*Bifidobacteria, Akkermansia, Shewanella, Allobaculum,* *Peptostreptococcaceae*, *Intestinibacter*	Li et al. (2019)
		down	*Prevotella, Deferribacteres*	
HFD Sprague-Dawley rats	16S rRNA	up	*Lactobacillus, Roseburia, Akkermansia*	Cui et al. (2019)
		down	*Desulfovibrio,* *Lachnospiraceae* *NK4A136*	
HFD Wistar rats	qPCR	up	*Lactobacillus* spp.*, Bifidobacterium* spp.*,*	Khat-Udomkiri et al. (2020)
		down	*Escherichia* spp.*, Clostridium perfringens*	
T2DM patients	Metagenomic	up	*Escherichia* spp.	Forslund et al. (2015)
		down	*Intestinibacter* spp.	
T2DM patients	16S rRNA	up	*Butyrivibrio, Bifidobacterium bifidum, Megasphaera, Prevotella, Akkermansia muciniphila*	de la Cuesta-Zuluaga et al. (2017)
		down	*Oscillospira, Barnesiellaceae,* Clostridiaceae *02d06*	
T2DM patients	Metagenomic	up	*Akkermansia muciniphila, Pectobacterium, Pantoea, Serratia, Raphidiopsis, Dickeya, Helicobacter, Shewanella, Erwinia, Cronobacter, Rheinheimera, Dermacoccus, Yersinia, Bacillus, Pseudomonas, Salmonella, Klebsiella, Enterobacter, Citrobacter, Escherichia*	Wu et al. (2017a)
		down	*Dethiosulfovibrio, Deferribacter, Bartonella, Acetivibrio, Hippea, Pseudogulbenkiania, Pseudoflavonifractor, Subdoligranulum, Intestinibacter*	
T2DM patients	16S rRNA	up	*Akkermansia, Clostridium cluster XIVa, Clostridium cluster XIVb, Escherichia/Shigella, Klebsiella, unclassified* *Enterobacteriaceae*	Hiel et al. (2020)
		down	*Clostridium cluster XI, Clostridium cluster XVIII, Roseburia, unclassified* *Lachnospiraceae*	

Abbreviations: HFD, high-fat diet; T2DM, type 2 diabetes mellitus. All the bacterial taxa are written in italics.

Metformin can influence the gut microbiota composition by directly affecting bacterial growth and altering the intestinal environment. It boosts the development of bacteria that generate short-chain fatty acids (SCFAs) in the gut of people with T2DM, leading to an increase in SCFAs levels in the colon, ultimately improving host metabolism (Forslund et al., 2015; Zhang et al., 2015; Wu et al., 2017a; de la Cuesta-Zuluaga et al., 2017; Mueller et al., 2021). Notably, elevated levels of SCFAs enhance glucose management through processes such as activation of intestinal gluconeogenesis and increased GLP-1 secretion, this promotes metabolic benefits in terms of energy balance (Cani et al., 2009; De Vadder et al., 2014; Chambers et al., 2015; Forslund et al., 2015). Propionate has been extensively characterized as a substrate for hepatic gluconeogenesis (Huntington, 1990). Research indicates that propionate undergoes conversion to glucose through intestinal gluconeogenesis prior to reaching the liver, conferring metabolic advantages in maintaining energy equilibrium (De Vadder et al., 2014). This is evidenced by reduced adiposity and body weight, notwithstanding similar levels of food consumption, alongside enhanced glucose regulation, marked by a reduction in hepatic glucose output. Consequently, SCFAs hold promise for glycemic control by modulating intestinal gluconeogenesis. Furthermore, metformin diminishes the presence of *Bacteroides fragilis*, a bacterium that suppresses bile salt hydrolase activity, leading to a rise in secondary bile acids like GUDCA. This inhibits the activation of intestinal FXR signaling, thereby facilitating enhanced glucose homeostasis (Sun et al., 2018).

Changes in the gut microbiota caused by metformin are crucial for its ability to reduce inflammation. Recent studies indicate that metformin strengthens the protective function of the intestinal mucosal barrier by increasing the abundance of *Akkermansia muciniphila* and the number of goblet cells, resulting in a thicker mucus layer that reduces inflammation in the intestines (Shin et al., 2014; Zhou et al., 2016; Ahmadi et al., 2020; Ke et al., 2021). Additionally, metformin boosts the relative presence of *Lactobacillus* spp. and *Akkermansia* spp., effectively moderating the microbial imbalance and colonic inflammation triggered by experimental colitis, alongside preserving the integrity of the mucus barrier (Ke et al., 2021; Liu et al., 2021).

Metformin’s impact on the gut microbiome also plays a role in metformin’s ability to fight tumors. Metformin administered orally, as opposed to injected intraperitoneally, inhibited tumor growth in mice fed a high-fat diet (Broadfield et al., 2022). Moreover, the transmission of gut bacteria from mice given metformin to other mice significantly slowed down the advancement of tumors, which was associated with an increased amount of bacteria that produce SCFAs and a decrease in the activity of genes necessary for making cholesterol in the tumor (Broadfield et al., 2022). Additionally, metformin extended the lifespan of *C. elegans* in co-culture with *Escherichia coli*, possibly due to the gut microbiota-derived metabolite agmatine (Pryor et al., 2019). The connection between metformin’s lifespan-extending effects in T2DM patients and increased agmatine levels remains speculative.

Most clinical observations regarding metformin’s influence on gut microbiota predominantly focus on alterations in fecal microbiota (Forslund et al., 2015). Nevertheless, metformin also impacts the microbiome in the small intestine, causing distinct changes in the microbiota of the duodenum, jejunum, and ileum in rodents (Bailey et al., 2008; Bauer et al., 2018; Bravard et al., 2021). The alterations in the microbial makeup of the small bowel align with fluctuations in the activity of genes associated with absorbing glucose and fatty acids in the intestines, amplifying the positive metabolic impacts of metformin (Bauer et al., 2018; Sun et al., 2018). Therefore, the microbiota throughout various sections of the gastrointestinal tract may play distinct roles.

### 2.4 Metformin and drug transporter proteins

Metformin exhibits a relatively modest oral bioavailability, ranging from 50% to 60% (Pentikäinen et al., 1979). Isotopic tracer techniques have shown that most of the ^14^C-labeled metformin is absorbed in the small intestine, with 20% being absorbed in the duodenum and 60% in the jejunum and ileum combined (Vidon et al., 1988; Dawed et al., 2019). Most of the metformin is eliminated in the urine without being changed by metabolic processes, with the rest being removed through the faeces (Tucker et al., 1981). Metformin, being a molecule with high affinity for water, usually remains in a positively charged state with a proton under normal bodily conditions (Foretz et al., 2019). Its absorption is dose-responsive and subject to saturation (Tucker et al., 1981; Proctor et al., 2008), indicating reliance on specific transporter proteins for its uptake.

Transporter proteins are crucial in the pharmacokinetic dynamics of medications, significantly influencing their effectiveness, side effects, and toxicity. Changes in the performance of these proteins have a direct effect on how the drug is absorbed, distributed, metabolized, and eliminated (Nies et al., 2008). Studies conducted with Caco-2 cells in a laboratory setting, utilizing inhibitors of transporter proteins and knockout methods, have pinpointed the main transporter proteins responsible for metformin as the organic cation transporters (OCTs), plasma membrane monoamine transporter (PMAT), serotonin transporter (SERT), and high-affinity choline transporter (CHT) (Han et al., 2015). Moreover, the multidrug and toxin extrusion proteins (MATEs) are crucial in the transportation of metformin (Jensen et al., 2016). Notably, OCT1, PMAT, and SERT are the principal transporters facilitating metformin’s intestinal absorption in intestinal cells, contributing to 25%, 20%, and 20% of its transport, respectively (Han et al., 2015).

Research has shown that variations in the OCT1 gene play a significant role in the varying effectiveness of metformin treatment for individuals with T2DM (Gambineri et al., 2010; Umamaheswaran et al., 2015; Sundelin et al., 2017; Chan et al., 2018). However, metformin extended-release preparations are absorbed into the bloodstream through intestinal transit proteins in about 50% of the amount of the regular preparations, but they have similar glucose-lowering effects compared to the regular preparations (Buse et al., 2016). Even under equal dosage conditions, the metformin extended-release formulation may be more effective (Buse et al., 2016). In line with this finding, a different research discovered that changes in the *SLC22A1* gene, responsible for OCT1, decreased the absorption of metformin in the liver without impacting the ability of metformin to lower glucose levels in individuals with T2DM (Zhou et al., 2009; Dujic et al., 2017). While OCT1 transporter proteins are involved in the pharmacokinetics of metformin, their impact on the effectiveness of metformin is currently a topic of debate.

### 2.5 Modulation of immune response

The immune system plays an essential role in developing and progressing many diseases. The gut microbiota and its byproducts play a vital role in the growth and upkeep of immune cells within the digestive system (Postler and Ghosh, 2017). Metformin can impact the body’s immune system by changing the gut microbiota and its metabolites, which may boost the inflammatory reaction (Postler and Ghosh, 2017). Research using mouse models of obesity and T2DM demonstrated that an increase in *A. muciniphila* following metformin treatment in obese mice activated regulatory T-cells in visceral adipose tissue. The activation resulted in decreased levels of pro-inflammatory cytokines IL-1β and IL-6 in adipose tissue, ultimately reducing inflammation (Shin et al., 2014). Additionally, metformin has demonstrated anti-inflammatory effects through the inhibition of IL-18 expression, which is linked to alterations in the intestinal microbiome (Lee et al., 2019). Another study found that treatment with metformin can regulate the immune response in the intestines of mice by altering the composition of the gut microbiome, particularly through the downregulation of the NF-κB signaling pathway (Brīvība et al., 2023). Therefore, metformin potentially enhances immune responses by modulating the gut microbiota.

Metformin has been shown to have some anti-inflammatory effects in clinical studies in patients with T2DM, in experimental studies in rodent models of obesity and T2DM, and *in vitro* experiments with several immune cells (Foretz et al., 2019; Kristófi and Eriksson, 2021). Metformin reduced colonic mucosal damage in a rat model of colitis by addressing oxidative stress and inhibiting the NF-κB-mediated inflammatory signaling pathway (Pandey et al., 2017). Changes in the local microenvironment within metabolic organs have a significant impact on the function of tissue-resident and newly recruited macrophages, leading to metabolic inflammation linked to obesity (Wculek et al., 2022). And *in vitro*, metformin reduces the inflammatory activation of macrophages by interfering with the metabolic changes linked to inflammation (Xiong et al., 2021). Metformin reduces the synthesis of pro-inflammatory cytokines in macrophages at a molecular level by blocking the transition from monocytes to macrophages via the AMPK-STAT3 pathway (Vasamsetti et al., 2015). Additionally, it suppresses inflammation by blocking the synthesis of endogenous fatty acids and its associated palmitoylation in macrophages (Xiong et al., 2021).

Persistent mild inflammation and changes in the immune system are characteristic features of the aging process and diseases associated with old age (Franceschi et al., 2018; Aiello et al., 2019). Studies suggest that metformin could impact the longevity of mice and *C. elegans* by regulating the mTOR signaling pathway and suppressing the release of CCL11 cytokines, which are connected to age-related cellular and tissue impairments (Thakkar et al., 2013; Bannister et al., 2014; Wu et al., 2016; Howell et al., 2017; Ivanovska et al., 2020). Surprisingly, metformin prolonged the lifespan of mammals and decreased lung inflammation caused by COVID-19 in a mouse model of acute respiratory distress, possibly by blocking the activation of NLRP3 inflammasome through the inhibition of mitochondrial DNA synthesis (Xian et al., 2021). Despite these discoveries, the relationship between them and the mechanisms of action within the gastrointestinal tract has not been clearly established, suggesting that further research in this area is warranted.

### 2.6 Others

The crosstalk between the gut and other organs can also facilitate the hypoglycemic effect of metformin. For instance, metformin can achieve its glucose-lowering effects through the gut-liver axis (Tobar et al., 2023). A recent PET-CT study revealed that metformin enhances glucose uptake in the basolateral area of the intestines, leading to the production of lactate (reducing pH and bicarbonate in portal vein) and acetate that travel to the liver via the portal vein and lower hepatic glucose production (Tobar et al., 2023). By this mechanism, metformin promotes gut-liver crosstalk, which further affects the regulation of glucose levels. While the results of this article do not exclude a direct action of metformin in the liver, they suggest that the first site of metformin action is the gut, via gut-hepatic crosstalk, and that it may play a role in the control of hepatic glucose production, integrating the site and mechanism of metformin action.

Metformin can also exert its glucose-lowering effects through a gut-liver-brain axis (Duca et al., 2015). The researchers demonstrated that intraduodenal infusion of metformin activated duodenal mucosal AMPK and lowered hepatic glucose production (HGP) in rat models of insulin resistance induced by a high-fat diet. They found that this effect was mediated through a complex inter-organ crosstalk involving the gut-brain-liver axis. Specifically, the lowering of HGP by metformin required the activation of duodenal glucagon-like peptide-1 receptor (GLP-1R) - protein kinase A (PKA) signaling and a neuronal-mediated pathway connecting the gut and liver via the brain. The researchers also found that the HGP-lowering effect of metformin could be negated by locally injecting a GLP-1 receptor antagonist into the duodenum, supporting the importance of local activation of the GLP-1 receptor in the duodenum for the glucose-lowering action of metformin. This emphasizes the role of the duodenal GLP-1 receptor in metformin’s action through the gut-liver-brain axis to lower HGP. Therefore, metformin can achieve glucose-lowering effects via the gut-liver axis and the gut-liver-brain axis, but the first site of metformin action is still the gut, so the gastrointestinal tract remains the main target of metformin action.

## 3 Adverse effects

### 3.1 Gastrointestinal effects

Despite its widespread availability and affordability, metformin is associated with gastrointestinal side effects, including nausea, vomiting, diarrhea, and bloating, affecting around 20% of patients (Sanchez-Rangel and Inzucchi, 2017; Zhang et al., 2023). Approximately 5% of individuals stopped taking the medication because of negative reactions, and easing symptoms may be achieved by slowly decreasing the dosage (Sanchez-Rangel and Inzucchi, 2017; Schommers et al., 2017). Recent research has identified the gut microbiota as the primary factor behind the gastrointestinal side effects of metformin (Bonnet and Scheen, 2017; Zhang et al., 2023). Excessive gas production by *E. coli* may be linked to gastrointestinal discomfort in patients following metformin consumption (Forslund et al., 2015). Research on the traits of gut microbiota in T2DM patients who are either tolerant or intolerant to metformin showed that alterations in gut microbiota composition following metformin therapy play a role in drug intolerance (Díaz-Perdigones et al., 2022). Among the patients who developed gastrointestinal side effects, the number of gut bacteria such as *Sutterella*, *Allisonella*, *Akkermansia*, *Bacteroides*, and *Paraprevotella* species was significantly elevated (Bryrup et al., 2019), which may be a possible cause of gastrointestinal discomfort reactions in some patients. Thus, gastrointestinal adverse effects may be related to metformin-induced changes in the gut microbiota.

Variants of the drug transporter protein gene could potentially contribute to gastrointestinal side effects in specific patients who are prescribed metformin. Metformin was discovered to be taken up by transporter proteins on the apical membrane surface of small intestinal epithelial cells in a study conducted on Caco-2 cells. However, only a small amount of metformin was transported through the outer basement membrane of the small intestinal epithelial cells into the bloodstream, resulting in metformin accumulation in these cells (Proctor et al., 2008). Therefore, high localized concentrations of metformin may have contributed to the consequence of side effects such as gastrointestinal distress. A different research project found that genetic variations in the OCT1, PMAT, and SERT transporter proteins were linked to a higher likelihood of gastrointestinal side effects in patients who cannot tolerate metformin (Dujic et al., 2015; Dawed et al., 2019). Additionally, taking medications that inhibit these transporter proteins also raised the risk of experiencing gastrointestinal discomfort (Dawed et al., 2019). Therefore, the cause of the emergence of gastrointestinal distress after taking metformin may also be related to mutations in the patient’s drug transporter protein genes.

### 3.2 Lactic acidosis

During metformin treatment, plasma lactate concentration increases with enhanced glucose uptake in the intestine, resulting in side effects in some patients with one or more risk factors for lactic acidosis (Bailey et al., 1992; Misbin et al., 1998; Davis et al., 2001). Metformin-induced lactate production in rats was found to be most concentrated in the hepatic portal vein, accompanied by a decrease in glucose levels, indicating that the gastrointestinal tract is the primary location for glucose consumption and lactate generation following metformin administration (Bailey et al., 1992). This was further verified in other experiments. For example, a 10% increase in lactic acid concentration in the intestines of rats following intestinal injection of metformin (Bailey et al., 1992). Similarly, cells cultured in a culture medium containing high concentrations of metformin generated more lactic acid by *in vitro* cell culture experiments (Bailey et al., 1992). Furthermore, elevated levels of metformin suppressed the activity of mitochondrial respiratory chain complex Ⅰ (Owen et al., 2000; Schommers et al., 2017) and modified the expression of genes related to mitochondria (Yang et al., 2021), potentially leading to a rise in anaerobic glucose metabolism. Nevertheless, an excessive metformin dosage could result in lactic acidosis and additional adverse reactions in individuals with one or more risk factors for lactic acidosis (Misbin et al., 1998). Metformin toxicity can be categorized into metformin-associated lactic acidosis (MALA), metformin-induced lactic acidosis (MILA), and metformin-unrelated lactic acidosis (MULA) (Rivera et al., 2023). Of these, MALA is the rare and most severe form, with a mortality rate of up to 50%. Therefore, in the ED, the types of lactic acidosis should be differentiated and managed separately according to the patient’s usual dose of metformin.

### 3.3 Vitamin B12 deficiency

Several case reports have now confirmed that metformin can cause vitamin B12 malabsorption as a potential side effect. In a study with 256 participants, 19 patients who were given metformin had vitamin B12 deficiency (<150 pmol/L), while five in the placebo group did. Additionally, 35 in the metformin group had low vitamin B12 levels (150–220 pmol/L), compared to 13 in the placebo group (de Jager et al., 2010). Metformin may lead to a deficiency in vitamin B12 by impacting the absorption mechanism of vitamin B12 in the cells of the small intestine (Sayedali et al., 2023). In diabetic patients, a lack of vitamin B12 in the future can lead to or speed up the development of distal symmetrical and autonomic neuropathy (Bell, 2022). Hence, it is advisable for individuals on long-term metformin therapy to undergo routine monitoring of their vitamin B12 levels (Langan and Goodbred, 2017).

## 4 Conclusion

**FIGURE 1:**
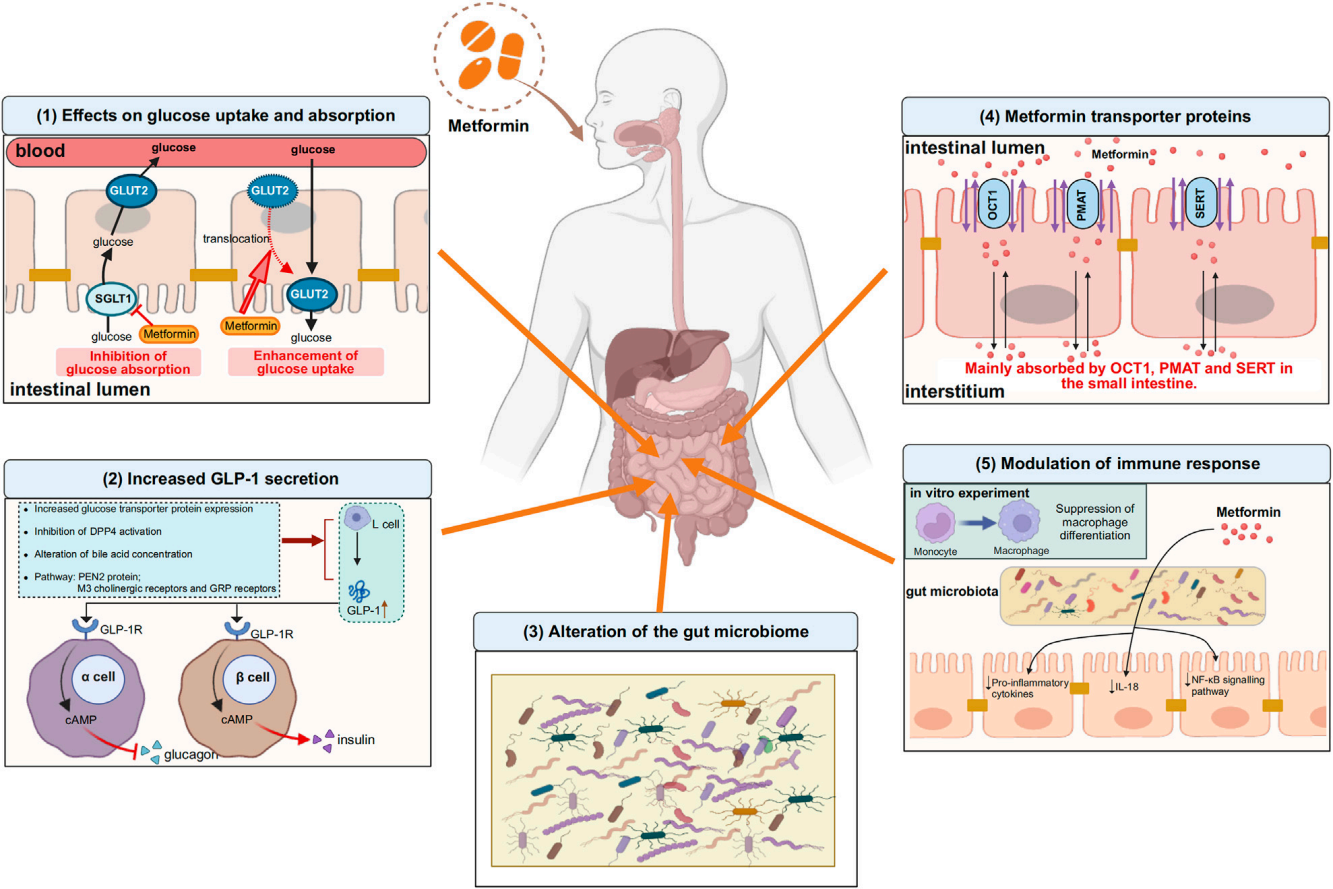
Some of the actions of metformin in the gastrointestinal tract. (1) Effects on glucose uptake and absorption. On the one hand, metformin inhibits the absorption of glucose from the intestinal lumen into the bloodstream by decreasing the expression of SGLT1 on the apical membrane of enterocytes, and on the other hand, metformin translocates GLUT2, which is located on the basolateral membrane of enterocytes, to the apical membrane and promotes glucose uptake from the blood into the intestinal lumen. (2) Increased GLP-1 secretion. Metformin promotes GLP-1 secretion from L cells by increasing glucose transporter protein expression, inhibiting DPP4 activation, regulating bile acid concentration, and mediating PEN2 protein, M3 cholinergic receptors and GRP receptors, after which GLP-1 binds to GLP-1 receptors on pancreatic islet cells and increases cAMP levels, followed by glucose regulation through inhibition of glucagon secretion and enhancement of insulin secretion. (3) Alteration of the gut microbiome. Gut microbiome is altered with metformin use. (4) Metformin transporter proteins. Metformin is absorbed into the interstitium mainly via OCT1, PMAT and SERT in the small intestine. (5) Modulation of immune response. Metformin can modulate intestinal immune responses by altering the composition of the gut microbiota, particularly through the suppression of pro-inflammatory cytokines and the downregulation of the NF-κB signaling pathway, thereby achieving immunomodulation. *In vitro* experiments, metformin inhibited monocyte to macrophage differentiation. The upward arrows indicate increases, the downward arrows indicate decreases. SGLT1, sodium-glucose transporter 1; GLUT2, glucose transporter 2; GRP: gastrin-releasing peptide; GLP-1, glucagon-like peptide-1; DPP4, dipeptidyl peptidase-4; GLP-1R, glucagon-like peptide-1 receptor; cAMP, cyclic adenosine monophosphate; OCT1, organic cation transporter 1; PMAT, plasma membrane monoamine transporter; SERT, serotonin transporter; NF-κB, nuclear factor kappa-B. Image created with BioRender.com, with permission.

Metformin, a widely prescribed medication, has been in use for more than six decades on a global scale. However, the mechanism of its therapeutic action is not fully understood. In addition to treating T2DM, metformin has also been found to be effective in various diseases in the clinical process, such as obesity, cancer, cardiovascular disease, and other inflammation-related diseases. It can also reduce the development of Long COVID and even protect against aging. While the liver was previously thought to be the main target of metformin’s glucose-lowering effects, recent evidence suggests that other parts of the body, such as the gastrointestinal tract, also play a role in its overall clinical advantages. Research has discovered that metformin can achieve its glucose-lowering effect through the gut-liver axis and the gut-liver-brain axis. However, the initial site of metformin’s action remains the gut, leading us to believe that the gastrointestinal tract is the primary target of metformin’s action. In the gastrointestinal tract, current studies have identified that metformin effects glucose uptake and absorption, influences GLP-1 secretion, modulates immune homeostasis, and regulates the gut microbiota, detailed information was drawn in Figure 1. The intestines are the second biggest organ that uses glucose, following the brain. This means that the primary way metformin helps regulate glucose levels is perhaps by directly affecting how glucose is processed in the intestines.

Some people may experience side effects after taking metformin, with the main symptoms being gastrointestinal discomforts such as nausea, vomiting, diarrhea, bloating, and in a few cases, lactic acidosis, and vitamin B12 malabsorption. Side effects vary individually, and the reasons may be related to the high local concentration of metformin in the gastrointestinal tract and the gut microbiota. The intestinal microecology is complex and studying the mechanisms underlying metformin treatment is challenging. Examining the impact of metformin on the gastrointestinal tract will enhance our comprehension of how metformin works.
